# Updates to the recently introduced family *Lacipirellulaceae* in the phylum *Planctomycetes*: isolation of strains belonging to the novel genera *Aeoliella*, *Botrimarina*, *Pirellulimonas* and *Pseudobythopirellula* and the novel species *Bythopirellula polymerisocia* and *Posidoniimonas corsicana*

**DOI:** 10.1007/s10482-020-01486-3

**Published:** 2020-11-05

**Authors:** Sandra Wiegand, Mareike Jogler, Christian Boedeker, Anja Heuer, Stijn H. Peeters, Nicolai Kallscheuer, Mike S. M. Jetten, Anne-Kristin Kaster, Manfred Rohde, Christian Jogler

**Affiliations:** 1 Institute for Biological Interfaces 5, Karlsruhe Institute of Technology, Eggenstein-Leopoldshafen, Germany; 2 Department of Microbiology, Radboud Universiteit, Nijmegen, The Netherlands; 3 Department of Microbial Interactions, Friedrich Schiller University, Jena, Germany; 4 Leibniz Institute DSMZ, Brunswick, Germany; 5 Central Facility for Microscopy, Helmholtz Centre for Infection Research, Brunswick, Germany

**Keywords:** Aquatic bacteria, Biotic surfaces, *Lacipirellula*, ‘*Bythopirellula goksoyri’*, *Pirellulales*, *Planctomycetia*

## Abstract

Eight novel strains of the phylum *Planctomycetes* were isolated from different aquatic habitats. Among these habitats were the hydrothermal vent system close to Panarea Island, a public beach at Mallorca Island, the shore of Costa Brava (Spain), and three sites with brackish water in the Baltic Sea. The genome sizes of the novel strains range from 4.33 to 6.29 Mb with DNA G+C contents between 52.8 and 66.7%. All strains are mesophilic (T_opt_ 24–30 °C) and display generation times between 17 and 94 h. All eight isolates constitute novel species of either already described or novel genera within the family *Lacipirellulaceae*. Two of the novel species, *Posidoniimonas polymericola* (type strain Pla123a^T^ = DSM 103020^T^ = LMG 29466^T^) and *Bythopirellula polymerisocia* (type strain Pla144^T^ = DSM 104841^T^ = VKM B-3442^T^), belong to established genera, while the other strains represent the novel genera *Aeoliella* gen. nov.*, Botrimarina* gen. nov., *Pirellulimonas* gen. nov. and *Pseudobythopirellula* gen. nov. Based on our polyphasic analysis, we propose the species *Aeoliella mucimassa* sp. nov. (type strain Pan181^T^ = DSM 29370^T^ = LMG 31346^T^ = CECT 9840^T^ = VKM B-3426^T^), *Botrimarina colliarenosi* sp. nov. (type strain Pla108^T^ = DSM 103355^T^ = LMG 29803^T^), *Botrimarina hoheduenensis* sp. nov. (type strain Pla111^T^ = DSM 103485^T^ = STH00945^T^, Jena Microbial Resource Collection JMRC), *Botrimarina mediterranea* sp. nov. (type strain Spa11^T^ = DSM 100745^T^ = LMG 31350^T^ = CECT 9852^T^ = VKM B-3431^T^), *Pirellulimonas nuda* sp. nov. (type strain Pla175^T^ = DSM 109594^T^ = CECT 9871^T^ = VKM B-3448^T^) and *Pseudobythopirellula maris* sp. nov. (type strain Mal64^T^ = DSM 100832^T^ = LMG 29020^T^).

**Electronic supplementary material:**

The online version of this article (10.1007/s10482-020-01486-3) contains supplementary material, which is available to authorized users.

## Introduction

Together with *Verrucomicrobia, Chlamydiae* and other sister phyla, the bacterial phylum *Planctomycetes* is part of the PVC superphylum (Wagner and Horn 2006). According to the current taxonomy, the phylum *Planctomycetes* comprises the phylogenetic classes *Planctomycetia*, *Phycisphaerae* and *Candidatus* Brocadiae. Members of the class *Planctomycetia* have been found to occur ubiquitously but are predominant in aquatic environments (Wiegand et al. 2018). They have been isolated from numerous algal surfaces (Bengtsson et al. 2012; Boersma et al. 2019; Bondoso et al. 2014, 2015, 2017; Kallscheuer et al. 2019a, d; Lage and Bondoso 2014; Peeters et al. 2019; Vollmers et al. 2017; Waqqas et al. 2020), on which they sometimes even dominate the microbial community (Bengtsson and Øvreås 2010; Wiegand et al. 2018). Planctomycetes are considered important players in global carbon cycling (Wiegand et al. 2018) as they possess the metabolic ability to degrade complex carbon substrates (Jeske et al. 2013; Lachnit et al. 2013). Planctomycetes are suspected to produce small bioactive molecules (Graca et al. 2016; Jeske et al. 2016; Waqqas et al. 2020). Recently, stieleriacines, one of the first small molecules elucidated from planctomycetes, were shown to be involved in alteration of community compositions in biofilms (Kallscheuer et al. 2020a). This finding might lead to an explanation as to why the cells can dominate algal surfaces without being outcompeted, despite their slow growth rates (Wiegand et al. 2018).

Members of the class *Planctomycetia* divide by budding, without employing otherwise essential divisome proteins such as the canonically used division ring-assembling FtsZ (Jogler et al. 2012; Kallscheuer et al. 2019d; Pilhofer et al. 2008). Most of the species seem to perform a lifestyle switch during their development, alternating between planktonic, motile swimmer cells and sessile mother cells (Gade et al. 2005). Their periplasm can be extremely enlarged and compartmentalised (Acehan et al. 2013; Boedeker et al. 2017), most likely to enable the digestion of internalised polysaccharides (Boedeker et al. 2017). Interestingly, Planctomycetes were only recently found to possess a peptidoglycan cell wall (Jeske et al. 2015; van Teeseling et al. 2015), which was also found in the closely related *Verrucomicrobia* (Rast et al. 2017).

The class *Planctomycetia* was recently reclassified and now comprises four different orders: *Pirellulales*, *Gemmatales*, *Isosphaerales* and the revised *Planctomycetales* (Dedysh et al. 2020). The latter three taxa harbour only a single family each, whereas the order *Pirellulales* currently consists of three families: *Pirellulaceae*, which contain most described genera and species (Kallscheuer et al. 2019b, c, d, 2020c; Rensink et al. 2020; Schubert et al. 2020); *Thermoguttaceae*, which feature the only known thermophilic Planctomycetes (Slobodkina et al. 2015, 2016); and *Lacipirellulaceae* (Dedysh et al. 2020). In addition to the type species of the type genus, *Lacipirellula parvula*, other described members of the family are ‘*Lacipirellula limnantha’* (Kallscheuer et al. in revision), ‘*Bythopirellula goksoyri’* (Storesund and Øvreås 2013) and *Posidoniimonas corsicana* (Kohn et al. 2020a).

In this study, we extend the current collection of *Lacipirellulaceae* strains by the description of eight novel species belonging to two known and four hitherto undescribed genera.

## Materials and methods

### Isolation of the novel strains and cultivation conditions

For strain isolation and cultivation, M1H NAG ASW medium was used. For medium preparation, 0.25 g peptone (Bacto), 0.25 g yeast extract (Bacto), 2.38 g (4-(2-hydroxyethyl)-1-piperazineethane-sulfonic acid) (HEPES) (10 mM), 250 mL artificial seawater (ASW) and 20 mL Hutner’s basal salt solution were mixed in a final volume of 973 mL double distilled water. The pH was adjusted to 7.5 using 5 M KOH and the solution was autoclaved for 20 min at 121 °C. After cooling, the following solutions were added aseptically: 1 mL of 25% (w/v) glucose, 5 mL vitamin solution, 1 mL trace element solution and 20 mL of a stock solution with 50 g/L *N*-acetyl glucosamine (NAG). ASW contained 46.94 g/L NaCl, 7.84 g/L Na_2_SO_4_, 21.28 g/L MgCl_2_·6 H_2_O, 2.86 g/L CaCl_2_·2 H_2_O, 0.384 g/L NaHCO_3_, 1.384 g/L KCl, 0.192 g/L KBr, 0.052 g/L H_3_BO_3_, 0.08 g/L SrCl_2_·6 H_2_O and 0.006 g/L NaF and was freshly prepared before addition to the base solution. Hutner’s basal salt solution was prepared by first dissolving 10 g nitrilotriacetic acid (NTA) in 700 mL double distilled water and adjusting the pH to 7.2 using 5 M KOH. Subsequently, the following compounds were added: 29.7 g MgSO_4_·7 H_2_O, 3.34 g CaCl_2_·2 H_2_O, 0.01267 g Na_2_MoO_4_·2 H_2_O, 0.099 g FeSO_4_·7 H_2_O and 50 mL metal salt solution 44. The solution was filled up to 1 L, sterilised by filtration and stored at 4 °C. Metal salt solution 44 consisted of 250 mg/L Na_2_-EDTA, 1095 mg/L ZnSO_4_·7 H_2_O, 500 mg/L FeSO_4_·7 H_2_O, 154 mg/L MnSO_4_·H_2_O, 39.5 mg/L CuSO_4_·5 H_2_O, 20.3 mg/L CoCl_2_·6 H_2_O and 17.7 mg/L Na_2_B_4_O_7_·10 H_2_O. In the first step, EDTA was dissolved and, if required, a few drops of concentrated H_2_SO_4_ were added to retard precipitation of the heavy metal ions. Metal salt solution 44 was sterilised by filtration and stored at 4 °C. Vitamin solution contained per litre: 10 mg *p*-aminobenzoic acid, 4 mg biotin, 20 mg pyridoxine hydrochloride, 10 mg thiamine hydrochloride, 10 mg calcium pantothenate, 4 mg folic acid, 10 mg riboflavin, 10 mg nicotinamide and 0.2 mg vitamin B_12_. *p*-Aminobenzoic acid was dissolved first and the solution was sterilised by filtration and stored in the dark at 4 °C. The trace element solution containing 1.5 g/L Na-nitrilotriacetate, 500 mg/L MnSO_4_·H_2_O, 100 mg/L FeSO_4_·7 H_2_O, 100 mg/L Co(NO_3_)_2_·6 H_2_O, 100 mg/L ZnCl_2_, 50 mg/L NiCl_2_·6 H_2_O, 50 mg/L H_2_SeO_3_, 10 mg/L CuSO_4_·5 H_2_O, 10 mg/L AlK(SO_4_)_2_·12 H_2_O, 10 mg/L H_3_BO_3_, 10 mg/L NaMoO_4_·2 H_2_O and 10 mg/L Na_2_WO_4_·2 H_2_O was sterilised by filtration and stored in the dark at 4 °C. The sampling locations and initial isolation of all strains are listed in Table S1. Sampled material was swabbed over or streaked on plates containing M1H NAG ASW medium solidified with 8 g/L gellan gum and additionally supplemented with 500 mg/L streptomycin, 200 mg/L ampicillin and 20 mg/L cycloheximide. The use of the two antibiotics and the anti-fungal agent cycloheximide is part of the enrichment strategy targeting strains of the phylum *Planctomycetes*, which are known to be resistant to both antibiotics. Colonies obtained after the first round of cultivation (four to six weeks) were streaked on a new plate and subsequently maintained in liquid M1H NAG ASW medium. Initial amplification and sequencing of the 16S rRNA gene was performed as previously described (Rast et al. 2017). This step was included to ensure that strains chosen for detailed characterisation are indeed members of the phylum *Planctomycetes*.

### Light microscopy

Phase contrast light microscopic analyses were performed employing a Nikon Eclipse Ti inverted microscope with a Nikon DS-Ri2 camera (blue LED). Specimens were immobilised in MatTek glass bottom dishes (35 mm, No. 1.5) employing a 1% (w/v) agarose cushion (Will et al. 2018). Images were analysed using the Nikon NIS-Elements software (version 4.3). To determine the cell size, at least 100 representative cells were counted manually (Annotations and Measurements, NIS-Elements) or by using the NIS-Elements semi-automated object count tool (smooth: 4×, clean: 4×, fill holes: on, separate: 4×). The Object Count tool enables setting a threshold for the image, automatically measures the binary objects and exports the measured data to a file.

### Electron microscopy

For field emission scanning electron microscopy, bacteria were fixed in 1% (v/v) formaldehyde in HEPES buffer (3 mM HEPES, 0.3 mM CaCl_2_, 0.3 mM MgCl_2_, 2.7 mM sucrose, pH 6.9) for 1 h on ice and washed once employing the same buffer (Rast et al. 2017). Cover slips with a diameter of 12 mm were coated with a poly-l-lysine solution (Sigma-Aldrich) for 10 min, washed in distilled water and air-dried. 50 µL of the fixed bacteria solution was placed on a cover slip and allowed to settle for 10 min. Cover slips were then fixed in 1% (v/v) glutaraldehyde in TE buffer (20 mM TRIS, 1 mM EDTA, pH 6.9) for 5 min at room temperature and subsequently washed twice with TE buffer before dehydrating in a graded series of acetone (10, 30, 50, 70, 90, 100%) on ice for 10 min. at each concentration. Samples from the 100% acetone step were brought to room temperature before placing them in fresh 100% acetone. Samples were then subjected to critical-point drying with liquid CO_2_ (CPD 300, Leica). Dried samples were covered with a gold/palladium (80/20) film by sputter coating (SCD 500, Bal-Tec) before examination in a field emission scanning electron microscope (Zeiss Merlin) using the Everhart–Thornley HESE2 detector and the in-lens SE detector in a 25:75 ratio at an acceleration voltage of 5 kV.

### Physiological analyses

For determination of the temperature optimum for growth, all strains were cultivated in M1H NAG ASW medium at pH 7.5 at different temperatures ranging from 10 to 40 °C. For determination of the pH optimum for growth, 100 mM HEPES was used instead of 10 mM for cultivations at pH 7.0, 7.5 and 8.0. For cultivation at pH 5.0, 5.5 and 6.0 HEPES was replaced by 100 mM 2-(*N*-morpholino)ethanesulfonic acid (MES), whereas 100 mM 3-(4-(2-hydroxyethyl)piperazin-1-yl)propane-1-sulfonic acid (HEPPS) was used as buffering agent at pH 8.5 and 100 mM *N*-cyclohexyl-2-aminoethanesulfonic acid (CHES) at pH 9.0, 9.5 and and 10.0. Cultivations for determination of the pH optimum were performed at 28 °C. Cell densities were determined as optical density at 600 nm (OD_600_).

### Genome information and genome-based analyses

The genomes of all strains were published previously (Wiegand et al. 2020) and are available from RefSeq under accession numbers SJPQ00000000 (strain Mal64^T^), CP036278 (strain Pan181^T^), SJPR00000000 (strain Pla108^T^), SJPH00000000 (strain Pla111^T^), SJPO00000000 (Pla123a^T^), SJPS00000000 (Pla144^T^), CP036291 (strain Pla175^T^) and CP036349 (strain Spa11^T^). The GenBank accession numbers of the respective 16S rRNA genes are MK554544 (strain Mal64^T^), MK559982 (strain Pan181^T^), MK554547 (Pla108^T^), MK554579 (strain Pla111^T^), MK554580 (strain Pla123a^T^), MK554548 (strain Pla144^T^), MK559987 (strain Pla175^T^) and MK554534 (strain Spa11^T^).

A genome-based analysis of enzymes participating in central carbon metabolism was conducted by examining locally computed InterProScan (Mitchell et al. 2019) results cross-referenced with information from the UniProt (UniProt 2019) database and BLASTp results of typical protein sequences. The analysis of the pan genomes was performed with anvi’o (Eren et al. 2015), following the pangenomics workflow (Delmont and Eren 2018).

### Construction of phylogenetic trees for the novel isolates

Maximum likelihood 16S rRNA gene sequence-based phylogeny was computed for the eight novel strains, the type strains of all recently described planctomycetal species (as of June 2020) (Kallscheuer et al. 2020b; Kumar et al. 2020; Peeters et al. 2020; Rivas-Marin et al. 2020a, b; Schubert et al. 2020) and all isolates recently published, but not yet described (Wiegand et al. 2020). The 16S rRNA gene sequences were aligned with SINA (Pruesse et al. 2012). The phylogenetic analysis was performed employing a maximum likelihood approach with 1000 bootstraps, the nucleotide substitution model GTR, gamma distribution and estimation of proportion of invariable sites (GTRGAMMAI option) (Stamatakis 2014). Three 16S rRNA genes of bacterial strains from the PVC superphylum (*Lentisphaera araneosa*, acc. no. ABCK01; *Kiritimatiella glycovorans*, acc. no. CP010904.1 and *Opitutus terrae*, acc. no. AJ229235.1) served as outgroup.

For the multilocus sequence analysis (MLSA) the unique single-copy core genome of all analysed genomes was determined with proteinortho5 (Lechner et al. 2011) with the ‘selfblast’ option enabled. The protein sequences of the resulting orthologous groups were aligned using MUSCLE v.3.8.31 (Edgar 2004). After clipping, partially aligned *C*- and *N*-terminal regions and poorly aligned internal regions were filtered using Gblocks (Castresana 2000). The final alignment of 900 ubiquitous genes with a combined length of 440,140 conserved amino acid residues was concatenated and clustered using FastTree (Price et al. 2010). The outgroup consisted of three genomes from strains of the families *Pirellulaceae* and *Planctomycetaceae* (*Bremerella volcania*, acc. no. CP036289.1; *Rubinisphaera brasiliensis*, acc. no. CP002546.1 and *Gimesia maris*, acc. no. CP042910.1).

### Analysis of phylogenetic markers

The *rpoB* nucleotide sequences (encoding the RNA polymerase β-subunit) were taken from publicly available genome annotations and the sequence identities were determined as described previously (Bondoso et al. 2013) using Clustal Omega (Sievers et al. 2011). Alignment and matrix calculation were performed only using those parts of the sequence that would have been sequenced with the previously described primer set (Bondoso et al. 2013). The average nucleotide identity (ANI) was calculated using OrthoANI (Lee et al. 2016). The average amino acid identity (AAI) was obtained with the aai.rb script of the enveomics collection (Rodriguez-R and Konstantinidis 2016) and the percentage of conserved proteins (POCP) was calculated as described before (Qin et al. 2014).

## Results and discussion

### Sampling at different locations in the Baltic Sea and the Mediterranean Sea

The strains Spa11^T^, Pla108^T^, Pla111^T^, Mal64^T^, Pla123a^T^, Pla175^T^, Pan181^T^ and Pla144^T^ were recently reported as novel isolates obtained during a large diversity-driven cultivation campaign targeting the phylum *Planctomycetes* (Wiegand et al. 2020). The eight strains were isolated from water or biofilms obtained from different aquatic sampling locations: a hydrothermal vent area offshore of Panarea island (Pan181^T^), phytoplankton from a public beach at Mallorca island (Mal64^T^) and seawater from the Costa Brava, Spain (Spa11^T^). Additionally, strains were recovered from wood or polyethylene particles incubated for two weeks at three different samplings spots with brackish water in or adjacent to the Baltic Sea (Pla108^T^, Pla111^T^, Pla123a^T^, Pla175^T^ and Pla144^T^). More details regarding the sampling spots and the isolation are provided in Table S1.

### Phylogeny

**Fig. 1:**
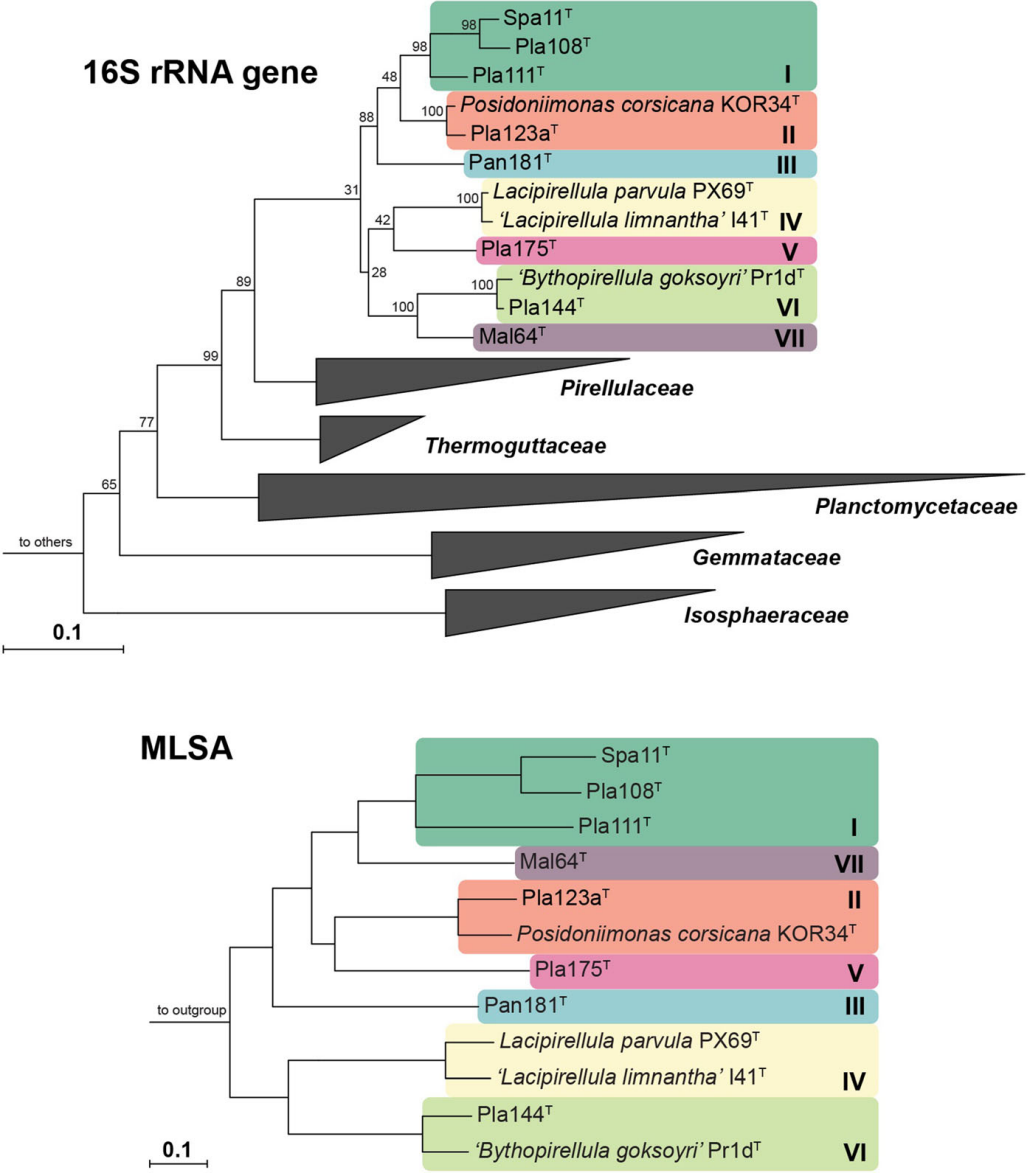
Phylogeny of the class *Planctomycetia* based on 16S rRNA gene sequence and of the family *Lacipirellulaceae* using whole genome-based multilocus sequence analysis (MLSA). Included in both analyses are the strains described here, ‘*Bythopirellula goksoyri’* (Storesund and Øvreås 2013), the recently described *Lacipirellula parvula* (Dedysh et al. 2020), ‘*L. limnantha’* (Kallscheuer et al. in revision) and *Posidoniimonas corsicana* (Kohn et al. 2020a). For the 16S rRNA gene sequence analysis, maximum likelihood estimation-gained bootstrap values after 1000 re-samplings are given at the nodes in %. The outgroup consists of three 16S rRNA genes from the PVC superphylum outside of the phylum *Planctomycetes*. Members of the other classes in the phylum *Planctomycetes* are not shown in the tree. For the MLSA-based phylogeny, reliability estimators based on Shimodaira-Hasegawa testing were determined. As they were always 1, they are not shown in the tree. The outgroup contains three genomes from the family *Planctomycetaceae*

Both 16S rRNA gene sequence as well as the MLSA-based phylogenetic trees suggest that all the strains described here are members of the recently established planctomycetal family *Lacipirellulaceae*, order *Pirellulales*, class *Planctomycetia*, phylum *Planctomycetes* (Dedysh et al. 2020) (Fig. 1). By closer examination of the phylogenetic trees, these strains and reference strains can be subdivided in seven groups: (I) strains Spa11^T^, Pla108^T^ and Pla111^T^; (II) *P. corsicana* KOR34^T^ (Kohn et al. 2020a) and strain Pla123a^T^; (III) strain Pan181^T^; (IV) *L. parvula* PX69^T^ (Dedysh et al. 2020) and ‘*L. limnantha*’ I41^T^ (Kallscheuer et al. in revision); (V) strain Pla175^T^; (VI) ‘*B. goksoyri’* Pr1d^T^ (Storesund and Øvreås 2013) and strain Pla144^T^, and (VII) strain Mal64^T^.

**Fig. 2:**
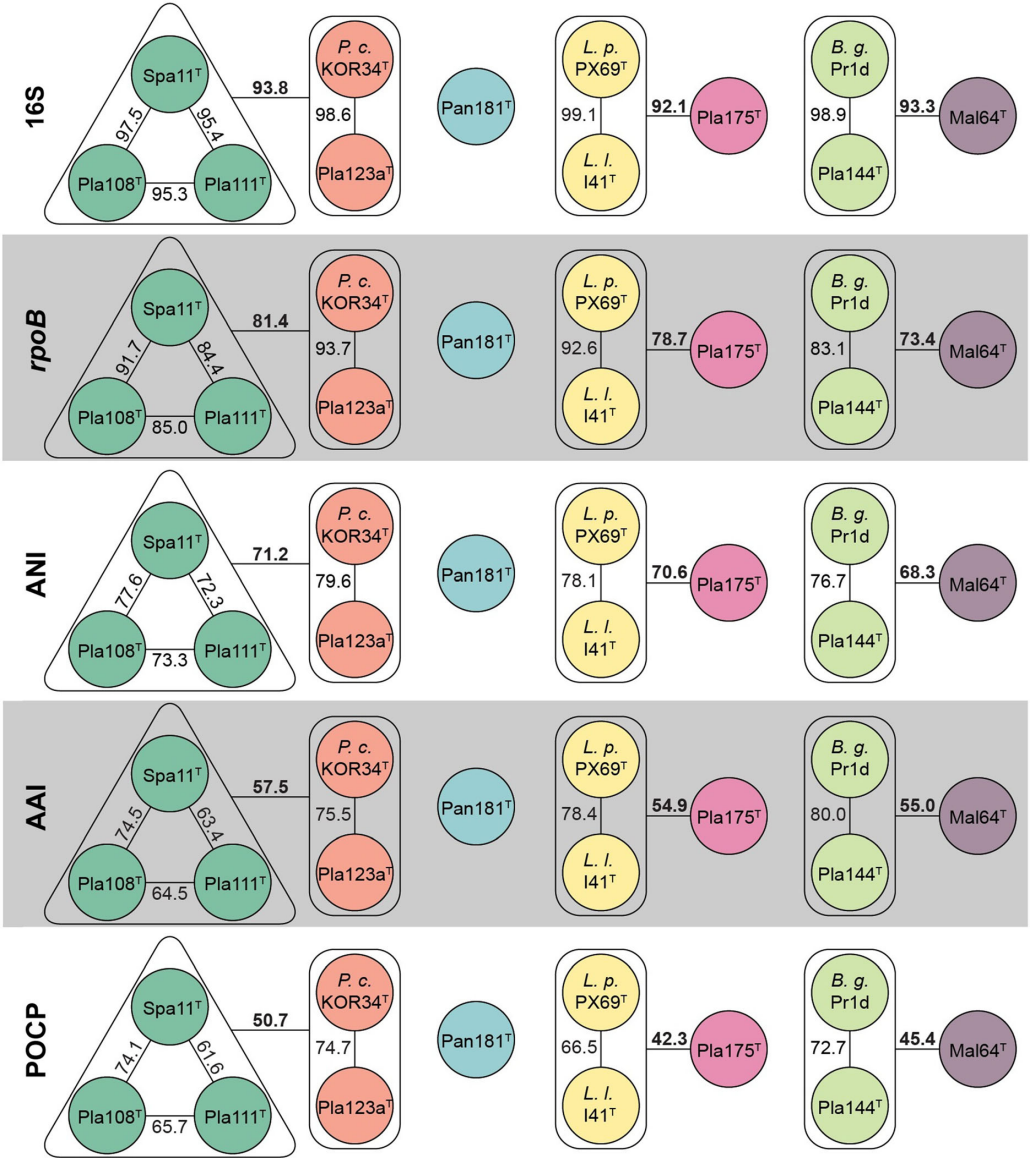
Analysis of phylogenetic markers for delineation of genera and species. Species suspected to belong to the same species (groups I to VIII) have the same colour: (I) green, (II) red, (III) blue, (IV) yellow, (V) hot pink, (VI) light green and (VII) purple. Values determined for average nucleotide identity (ANI) can be used to differentiate between species, while amino acid identity (AAI) and percentage of conserved proteins (POCP) allow to differentiate between genera. Values obtained for *rpoB* and 16S rRNA gene sequence identity are available for both applications. The values are given in black within groups and in black and bold between groups, where groups might be suspected to belong together. (Minimal) identity values between well resolved groups are not given in the figure but can be found in Tables S2–S6. *P. c.*: *Posidoniimonas corsicana*; *L. p.*: *Lacipirellula parvula; L. l.*: *‘Lacipirellula limnantha’*; *B. g.*: ‘*Bythopirellula goksoyri’*. (Color figure online)

Evaluation of ANI indicated that all the novel strains belong to separate species, given that all values (< 80%) are clearly below the threshold of 95% for delineation of prokaryotic species (Kim et al. 2014) (Fig. 2 and Table S2). This is also supported by identity of a 1200 bp partial sequence of the *rpoB* gene, for which the species threshold was proposed to be between 95.5 and 98.2% (Bondoso et al. 2013). For this phylogenetic marker, all strains turned out to have minimal identity values of < 93.7% (Fig. 2 and Table S3). For 16S rRNA gene sequence identities the situation is less clear, as some values are above the well-established species threshold of 98.7% (Yarza et al. 2014) (Fig. 2 and Table S4). This is a phenomenon known for the families *Planctomycetaceae* and *Pirellulaceae*, in which some novel species have been found to have a 16S rRNA gene sequence identity of up to 99.9% (Kohn et al. 2020b; Rensink et al. 2020). The subsequent analysis of the markers AAI and POCP indicated that the different groups (I-VII) belong to different genera within the family *Lacipirellulaceae*. With the exception of group I vs. VII (AAI: 60.4%; POCP: 57.6%) all minimal AAI values are below the proposed genus threshold of 60–80% (Luo et al. 2014) and POCP values are very close (< 50.7%) or below the genus threshold of 50% (Qin et al. 2014) (Fig. 2 and Tables S5, S6). The discrepancy between groups I and VII (strain Mal64^T^) is also reflected by the different positioning of strain Mal64^T^ in the phylogenetic trees (Fig. 1). However, the distinct clustering of the two groups in both the MLSA- and 16S rRNA sequence-based trees does not allow merging the two groups into one single genus. Moreover, the 16S rRNA gene sequence similarity between strain Mal64^T^ and group I strains is below the 94.5% threshold for delineation of prokaryotic genera as suggested by Yarza et al. (2014).

### Morphology and physiology

**Fig. 3:**
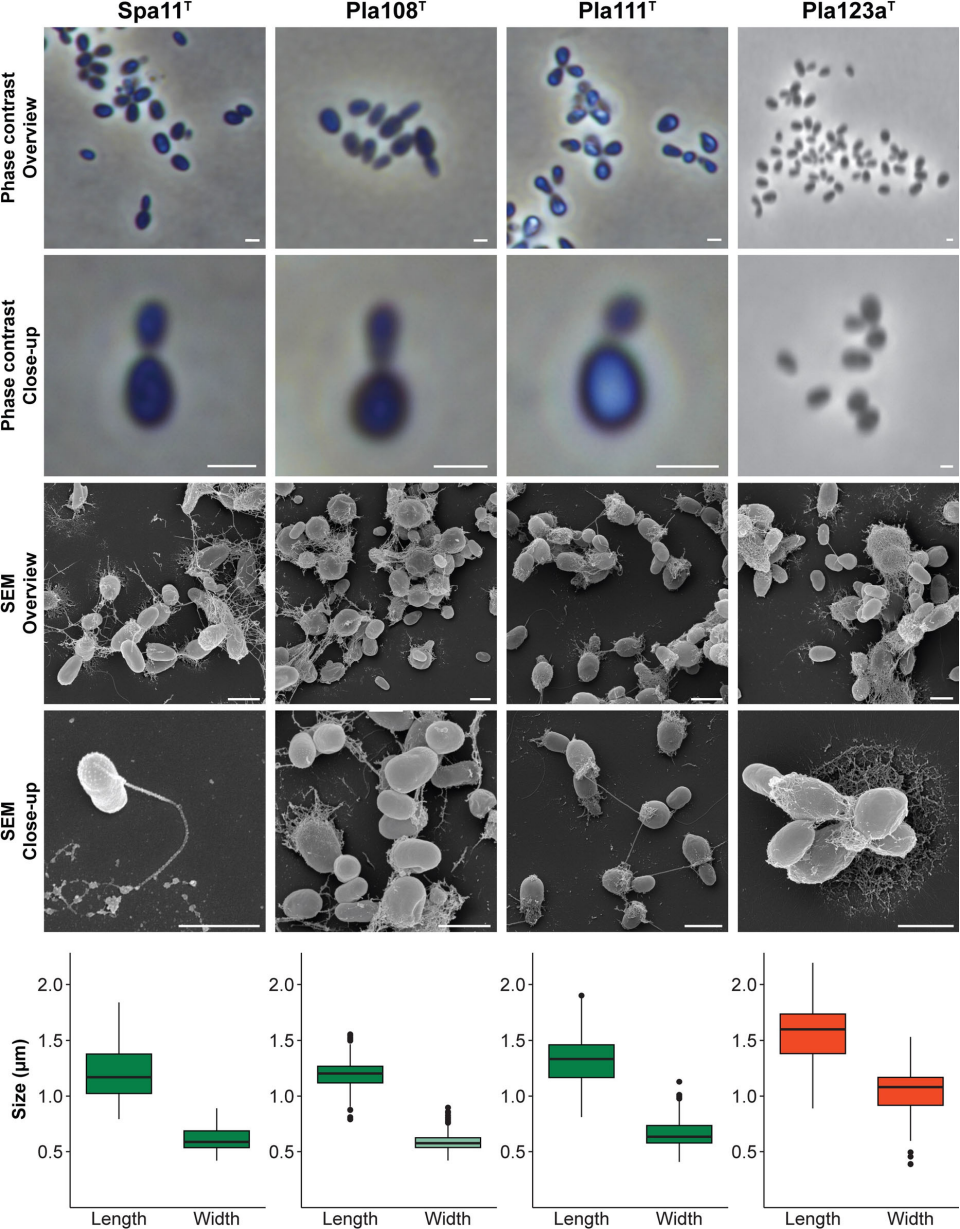
Microscopy images and cell size plot of novel strains of groups I (green) and II (red). The figure shows phase contrast as well as scanning electron microscopy (SEM) micrographs. The scale bar is 1 µm. For determination of the cell sizes at least 100 representative cells were counted manually or by using a semi-automated object count tool. Whiskers of box plots represent 1.5x interquartile range. (Color figure online)

**Fig. 4:**
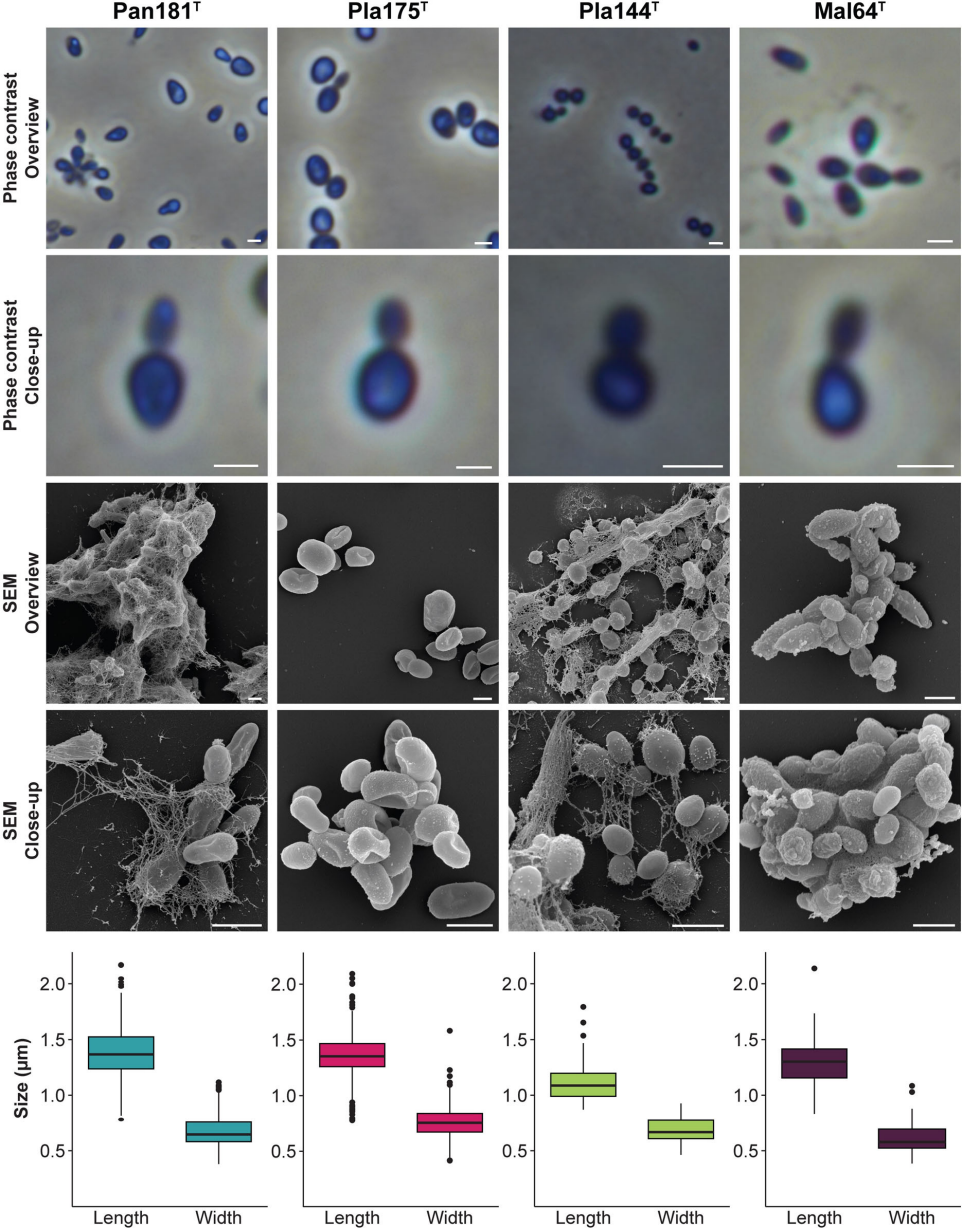
Microscopy images and cell size plot of novel strains of groups III (blue), V (hot pink), VI (light green) and VII (purple). The figure shows phase contrast as well as scanning electron microscopy (SEM) micrographs. The scale bar is 1 µm. For determination of the cell sizes at least 100 representative cells were counted manually or by using a semi-automated object count tool. Whiskers of box plots represent 1.5x interquartile range. (Color figure online)

Cell morphology and cell sizes of strains Spa11^T^, Pla108^T^, Pla111^T^, Pla123a^T^, Pan181^T^, Pla175^T^, Pla144^T^ and Mal64^T^ were determined by observation through light microscopy and scanning electron microscopy (SEM) using exponentially growing cultures (Figs. 3, 4). All strains form pear-shaped cells, with cell sizes given in Figs. 3 and 4 and Table S7. On average, they range from 1.1 to 1.6 µm in length and 0.6 to 1.1 µm in width. All strains divide by budding, with the released buds being shaped like the mother cells. For all strains with appropriate early exponential phase data, a dimorphic life cycle could also be observed (strains Spa11^T^, Pla111^T^, Pla123a^T^, Pla144^T^). In all strains, the cells are connected by short holdfast structures that are released opposite of the budding pole. This allows most of the strains to form larger loose aggregates, with the exception of strain Mal64^T^ (very dense aggregates) and Pla175^T^ (only very small aggregates). For all strains but the latter, the production of fibres, that seem to foster the formation of aggregates, could be observed at the budding pole. All strains possess crateriform structures, and, at least for strains Pla111^T^, Pla175^T^ and Pla144^T^, these structures are located at the budding pole. For most of the strains, flagella could be observed at some point of their life cycle. All strains are aerobic heterotrophs. The colonies of strains Spa11^T^, Pla123a^T^, Pan181^T^, Pla144^T^ and Mal64^T^ are non-pigmented, whereas the colonies of strains Pla108^T^, Pla111^T^, and Pla175^T^ are coloured in hot pink to red shades. The strains were found to grow over pH ranges of 5.0 to 9.0 with the optima being between pH 6.5 and 8.5 (Table 1, Fig. S1). While most of the strains are capable of growth in a broad spectrum within this range, strain Pla111^T^ only grew at pH 6.5–7.0. Growth of the examined strains was observed at temperatures between 10 and 39 °C with optimal growth between 24 and 30 °C (Table 1, Fig. S2). In this range, strain Pla144^T^ had the narrowest spectrum with growth only between 20 and 30 °C (T_opt_ of 27 °C). The growth rates at the optimal temperature in M1H NAG ASW medium were between 0.0074 and 0.041 h^−1^ which corresponds to doubling times between 17 and 94 h (Table 1).

**Table 1:** Ranges and optima for growth regarding pH and temperature and maximal growth rates of the novel strains

	pH	pH_opt_	T (°C)	T_opt_ (°C)	Growth rate at T_opt_ (h^−1^)	Generation time (h) at T_opt_
Spa11^T^	5.0–9.5	6.5	10–36	30	0.019	36.5
Pla108^T^	6.5–8.5	6.5	10–33	30	0.013	53.3
Pla111^T^	6.5–7.0	7.0	10–33	27	0.020	34.7
Pla123a^T^	6.0–8.5	7.5	10–33	27	0.030	23.1
Pan181^T^	5.5–9.0	7.5	10–39	28	0.033	21.0
Pla175^T^	5.5–8.5	6.5	10–33	24	0.0086	80.6
Pla144^T^	6.0–9.5	8.5	20–30	27	0.0074	93.7
Mal64^T^	5.5–9.0	7.0	15–36	27	0.041	16.9

Growth rates and generation times were both determined at the detected optimal growth temperature (T_opt_). The medium used was M1H NAG ASW, supplemented with different buffering agents for the determination of the pH range

### Genome-based analysis of enzymes participating in central carbon metabolism

The genomes of all these novel strains carry genes to allow the conversion of glucose to pyruvate via the Embden-Meyerhof-Parnas pathway, but probably not via the Entner-Doudoroff pathway (Table S8). For members of groups I, II, III, V and VI it is unsure if they are capable of performing gluconeogenesis, as they might lack a pyruvate carboxylase (EC 6.4.1.1) catalysing the formation of oxaloacetate from pyruvate (Table S8). As suggested by the presence of respective genes, all strains are capable of using the pentose phosphate pathway and also the tricarboxylic acid cycle appears to be functional. Interestingly, not all strains possess the 2-oxoglutarate dehydrogenase complex (members of groups I, VI and VII as well as one member of group II). However, all strains harbour genes coding for a putative 2-oxoacid:ferredoxin oxidoreductase, that also allows the oxidation of 2-oxoglutarate to succinyl-CoA (Table S8).

### Genomic features of the isolates

While the genomes of three of the strains characterised here are closed, the other genomes consist of up to 27 scaffolds (Table 2). The G+C content of the DNA varies between 58.0 and 66.5%, which brings the family *Lacipirellulaceae* into the upper quartile in terms of the G+C content of Planctomycetes (Table 2). The genome sizes of the strains fall between 4.33 and 6.62 Mb. Members of group I have the smallest genomes (Table 2), which are among the smallest genomes found in the class *Planctomycetia*. Most probably due to their smaller genomes, the number of hypothetical proteins is also relatively low throughout the family (38–44%), with a noticeable exception for *L. parvula* (66%). The strains have between 47 and 78 tRNAs and possess only one copy of the rRNA genes.

**Table 2:** Genomic features of the novel strains and other described strains of the family *Lacipirellulaceae*

	Genome size (Mb)	G+C content (%)	Genes (per Mb)	Protein-coding genes (per Mb)	Hypothetical proteins	Coding density (%)	tRNAs	Giant genes (> 15 kb)	Transposable elements
Spa11^T^	5.87 (1)	64.1	4653 (793)	4591 (782)	1923	85.8	54	1	5
Pla108^T^	5.27 (22)	65.4 ± 3.8	4226 (803)	4168 (792)	1701	87.0	53	1	13
Pla111^T^	4.33 (18)	63.1 ± 2.6	3479 (804)	3425 (792)	1307	87.5	47	2	5
*Posidoniimonas corsicana* KOR34^T^ (Kohn et al. 2020a)	6.77 (13)	66.7 ± 1.2	5344 (790)	5247 (776)	2219	86.5	91	2	3
Pla123a^T^	6.26 (27)	66.5 ± 3.2	4933 (788)	4871 (778)	2003	86.4	54	1	3
Pan181^T^	6.61 (1)	58.0	5414 (819)	5351 (810)	2315	86.3	52	0	3
*Lacipirellula parvula* PX69^T^ (Dedysh et al. 2020)	6.92 (1)	61.7	5665 (818)	5581 (806)	3702	84.7	73	1	9
‘*Lacipirellula limnantha’'* I41^T ^(Kallscheuer et al. in revision)	6.78 (1)	62.0	5568 (822)	5471 (807)	2570	83.5	85	0	11
Pla175^T^	6.62 (1)	66.5	5218 (788)	5137 (776)	2134	86.1	58	0	7
*’Bythopirellula goksoyri*’ Pr1d (Storesund and Øvreås 2013)	6.47 (1)	52.8	5395 (833)	5306 (820)	2322	86.5	68	0	8
Pla144^T^	6.14 (27)	52.9 ± 2.2	5134 (836)	5041 (821)	2188	86.7	78	0	8
Mal64^T^	5.07 (6)	66.2 ± 0.9	3953 (780)	3893 (768)	1561	85.2	52	1	2

Strain I41^T^ (Kallscheuer et al. in revision) is the only strain in the family known to carry a plasmid. However, data corresponding to the plasmid are not shown, but only reflect information based on the chromosome

**Fig. 5:**
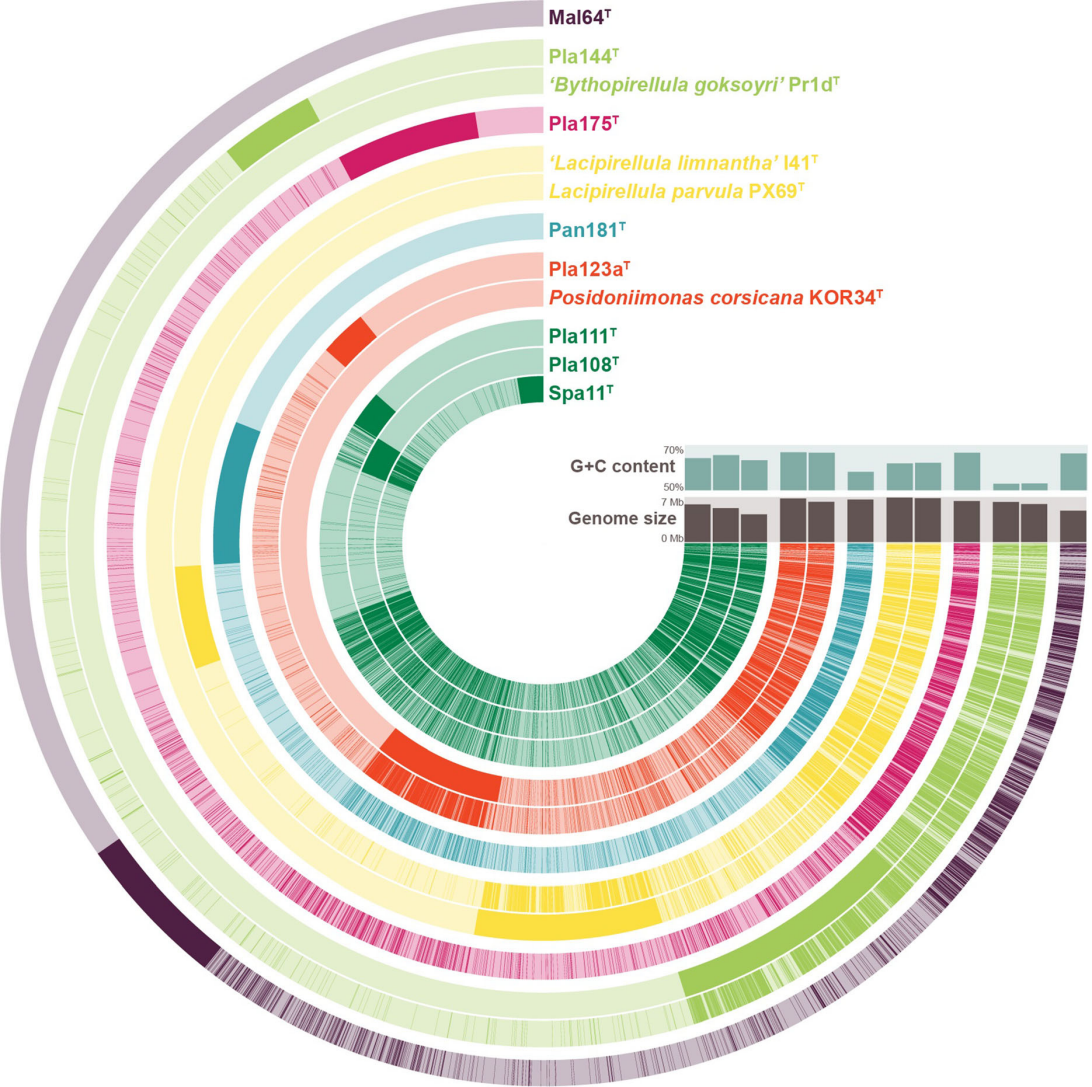
Pan genome of all strains within the family *Lacipirellulaceae*. Each open circle represents the pan genome of all strains, but is coloured darker when the gene is present in the respective genome. The colour represents the group affiliation of the genomes: (I) dark green, (II) red, (III) blue, (IV) yellow, (V) hot pink, (VI) light green and (VII) purple. The trees reflect the relatedness of the strains based on the absence/presence of genes. (Color figure online)

The determined phylogenetic groups I-VII can also be confirmed by the analysis of the pan genome of the strains (entirety of genes present in all genomes; Fig. 5). Next to genes present in all/many genomes (position 3 to 4 o’clock, Fig. 5), genes that are predominant in the separate groups support the phylogenetic assumptions: dense clusters can be found for all groups that contain more than one genome (II: red, 7 o’clock; IV: yellow, 6 o’clock;, VI: light green, 5 o’clock). At this stage, the only exception is again strain Mal64^T^ (VII, purple) that seems to cluster with members of group I (dark green) despite their distant relationship suggested by lower 16S rRNA gene sequence similarity.

Based on the phylogenetic analysis, the absence and presence of gene clusters as well as morphological and physiological data, we propose that each group (I to VII) corresponds to a separate genus within the family *Lacipirellulaceae*. On the same basis, we suggest that each member of each group belongs to a separate species. Therefore, we propose the following genus and species names: (I) *Botrimarina mediterranea* gen. nov., sp. nov. with type strain Spa11^T^, *Botrimarina colliarenosi* sp. nov. with type strain Pla108^T^, *Botrimarina hoheduenensis* sp. nov. with type strain Pla111^T^; (II) *Posidoniimonas polymericola* sp. nov. with type strain Pla123a^T^; (III) *Aeoliella mucimassa* gen. nov., sp. nov. with type strain Pan181^T^; (V) *Pirellulimonas nuda* gen. nov., sp. nov. with type strain Pla175^T^; (VI) *Bythopirellula polymerisocia* sp. nov. with type strain Pla144^T^ and (VII) *Pseudobythopirellula maris* gen. nov., sp. nov. with type strain Mal64^T^.

#### Emended description of the family *Lacipirellulaceae* (Dedysh et al. 2020)

The description is as given by Dedysh et al. 2020, with the following modification: The DNA G+C content of members of the genus ranges between 52 and 67%.

#### Description of the genus *Botrimarina* gen. nov.

*Botrimarina* (Bo.tri.ma.ri’na. L. fem. n. *botrus* a cluster of grapes; L. fem. adj. *marina* marine, of the sea; N.L. fem. n. *Botrimarina* a cluster of marine grapes, referring to the origin and the characteristics of the type strain of the type species).

Cells are pear-shaped and form aggregates. The DNA G+C content of the genomes of members of the genus ranges between 63 and 65%. The distinctiveness of the genus is confirmed by phylogenetic analysis. The type species is *Botrimarina mediterranea*. The genus belongs to the family *Lacipirellulaceae*, order *Pirellulales*, class *Planctomycetia*, phylum *Planctomycetes*.

#### Description of *Botrimarina mediterranea* sp. nov.

*Botrimarina mediterranea* (me.di.ter.ra’ne.a. L. fem. adj. *mediterranea* Mediterranean; corresponding to the origin of the strain from the Mediterranean Sea).

Cells are pear-shaped (size: 1.2 ± 0.2 × 0.6 ± 0.1 µm), form loose aggregates, divide by budding and feature a dimorphic life cycle. Cells have flagella, crateriform structures and fibres covering only one pole. On the opposite side to the latter, a holdfast structure is located. Cells are mesophilic and neutrophilic chemoorganoheterotrophs, growing over ranges of 10–36 °C (optimum 30 °C) and pH 5.0–9.5 (optimum 6.5). Colonies are white. The genome size of the type strain is 5.87 Mb with a G+C content of 64.1%.

The type strain is Spa11^T^ (= DSM 100745^T^ = LMG 31350^T^ = CECT 9852^T^ = VKM B-3431^T^), isolated from costal seawater at Costa Brava, Spain. The type strain genome (acc. no. CP036349) and 16S rRNA gene sequence (acc. no. MK554534) are available from GenBank.

#### Description of *Botrimarina colliarenosi* sp. nov.

*Botrimarina colliarenosi* (col.li.a.re.no.si. L. masc. n. *collis* a hill, heap; L. masc. adj. *arenosus* sandy; N.L. gen. n. *colliarenosi* from a sandy hill; corresponding to the origin of the type strain from Hohe Düne in Rostock, Germany).

Cells are pear-shaped (size: 1.2 ± 0.1 × 0.6 ± 0.1 µm), form aggregates and divide by budding. Cells have a holdfast structure, flagella, crateriform structures and polar fibres. Cells are mesophilic and neutrophilic chemoorganoheterotrophs, growing over a range of 10–33 °C (optimum 30 °C) and at pH 6.5–8.5 (optimum 6.5). Colonies are amaranth pink. The genome size of the type strain is 5.27 Mb with a DNA G+C content of 65.4 ± 3.8%.

The type strain is Pla108^T^ (= DSM 103355^T^ = LMG 29803^T^), isolated from wood particles placed in a small incubator, which was stored in a depth of two metres for 14 days close to the yacht harbour of Hohe Düne, a local district of Rostock, Germany. The type strain genome (acc. no. SJPR00000000) and 16S rRNA gene sequence (acc. no. MK554547) are available from GenBank.

#### Description of *Botrimarina hoheduenensis* sp. nov.

*Botrimarina hoheduenensis* (ho.he.due.nen’sis. N.L. fem. adj. *hoheduenensis* of Hohe Düne; corresponding to the origin of the type strain from Hohe Düne in Rostock, Germany).

Cells are pear-shaped (size: 1.3 ± 0.2 × 0.7 ± 0.1 µm), form loose aggregates, divide by budding and feature a dimorphic life cycle. Cells have flagella, crateriform structures and fibres, all emerging at the budding pole. A holdfast structure is located at the opposite pole. Cells are mesophilic and neutrophilic chemoorganoheterotrophs, growing over a range of 10–33 °C (optimum 27 °C) and at pH 6.5–7.0 (optimum 7.0). Colonies are carnation-pink pigmented. The genome size of the type strain is 4.33 Mb with a DNA G+C content of 63.1 ± 2.6%.

The type strain is Pla111^T^ (= DSM 103485^T^ = STH00945^T^, Jena Microbial Resource Collection JMRC), isolated from a biofilm formed in a small incubator, which in turn was stored in a depth of 2 metres for 14 days close to the yacht harbour of Hohe Düne, a local district of Rostock, Germany. The type strain genome (acc. no. SJPH00000000) and 16S rRNA gene sequence (acc. no. MK554579) are available from GenBank.

#### Emended description of the genus *Posidoniimonas* Kohn et al. (2020)

The description of the genus *Posidoniimonas* is as given previously (Kohn et al. 2020a) with the following modification: The G+C content of members of the genus is between 66.5 and 66.7%.

#### Description of *Posidoniimonas polymericola* sp. nov.

*Posidoniimonas polymericola* (po.ly.mer.i’co.la. N.L. neut. n. *polymerum* polymer; L. suff. *cola* an inhabitant, resident; N.L. fem. n. *polymericola* an inhabitant of polymers; corresponding to the origin of the type strain from polymeric material).

Cells are pear-shaped (size: 1.6 ± 0.3 × 1.0 ± 0.2 µm), form aggregates, divide by budding and feature a dimorphic life cycle. Cells have flagella, crateriform structures and polar fibres as well as a holdfast structure opposite to the fibre pole. Cells are mesophilic and neutrophilic chemoorganoheterotrophs, growing over a range of 10–33 °C (optimum 27 °C) and at pH 6.0–8.5 (optimum 7.5). Colonies are white. The genome size of the type strain is 6.26 Mb with a DNA G+C content of 66.5 ± 3.2%.

The type strain is Pla123a^T^ (= DSM 103020^T^ = LMG 29466^T^), isolated from wood particles placed in a small incubator, which was stored in a depth of 2 metres for 14 days below the pier of Heiligendamm (Seebrücke Heiligendamm), Germany. The type strain genome (acc. no. SJPO00000000) and 16S rRNA gene sequence (acc. no. MK554580) are available from GenBank.

#### Description of the genus *Aeoliella* gen. nov.

*Aeoliella* (Ae.o.li.el’la. N.L. fem. n. *Aeoliella* (dim. of L. fem. *aeolia*, the Eolian islands) a bacterium from the Eolian Islands, Italy).

Cells are pear-shaped and form aggregates. The distinctiveness of the genus is confirmed by phylogenetic analysis. The type species is *Aeoliella mucimassa.* The genus belongs to the family *Lacipirellulaceae*, order *Pirellulales*, class *Planctomycetia*, phylum *Planctomycetes*.

#### Description of *Aeoliella mucimassa* sp. nov

*Aeoliella mucimassa* (mu.ci.mas’sa. L. masc. n. *mucus* mucus, slime; L. fem. n. *massa* a lump; N.L. fem. n. *mucimassa* a mass of slime, corresponding to the characteristic of the cells to form very fibrous aggregates).

Cells are pear-shaped (size: 1.4 ± 0.3 × 0.7 ± 0.1 µm), form dense aggregates and divide by budding. Cells have crateriform structures and polar fibres. Cells are mesophilic and neutrophilic chemoorganoheterotrophs, growing over a range of 10–39 °C (optimum 28 °C) and at pH 5.5–9.0 (optimum 7.5). Colonies are white. The genome size of the type strain is 6.61 Mb with a DNA G+C content of 58.0%.

The type strain is Pan181^T^ (= DSM 29370^T^ = LMG 31346^T^ = CECT 9840^T^ = VKM B-3426^T^), isolated from the shallow sea hydrothermal vent system close to Panarea Island, Italy. The type strain genome (acc. no. CP036278) and 16S rRNA gene sequence (acc. no. MK559982) are available from GenBank.

#### Description of the genus *Pirellulimonas* gen. nov.

*Pirellulimonas* (Pi.rel.lu.li.mo’nas. N.L. fem. n. *Pirellula* name of a bacterial genus; L. fem. n. *monas* a unit, monad; N.L. fem. n. *Pirellulimonas* a monad of the order *Pirellulales*).

Cells are pear-shaped. The distinctiveness of the genus is confirmed by phylogenetic analysis. The type species is *Pirellulimonas nuda*. The genus belongs to the family *Lacipirellulaceae*, order *Pirellulales*, class *Planctomycetia*, phylum *Planctomycetes*.

#### Description of *Pirellulimonas nuda* sp. nov.

*Pirellulimonas nuda* (nu’da. L. fem. adj. *nuda* nude, bare; corresponding to the absence of matrix and fibers).

Cells are pear-shaped (size: 1.4 ± 0.2 × 0.8 ± 0.1 µm), form very small aggregates and divide by budding. Cells have crateriform structures located mainly at one pole. Cells are mesophilic and neutrophilic chemoorganoheterotrophs, growing over a range of 10–33 °C (optimum 24 °C) and at pH 5.5–8.5 (optimum 6.5). Colonies are ruby. The genome size of the type strain is 6.62 Mb with a DNA G+C content of 66.5%.

The type strain is Pla175^T^ (= DSM 109594^T^ = CECT 9871^T^ = VKM B-3448^T^), isolated from wood particles placed in a small incubator, which was stored in a depth of 2 metres for 14 days in the Unterwarnow, an estuary of the river Warnow close to Rostock, Germany. The sampling location was close to a discharge of a wastewater treatment plant. The type strain genome (acc. no. CP036291) and 16S rRNA gene sequence (acc. no. MK559987) are available from GenBank.

#### Emended description of the genus *Bythopirellula* (Storesund and Øvreås 2013)

The description is as given by Storesund and Øvreås 2013, with the following modifications: Oval cells occurring assembled in consortia or as single cells. Reproducing by budding, daughter cells are motile; mother cells are non-motile and attached to substrate or surface. Produce exopolysaccharide-like structures for attachment. Member of the family *Lacipirellulaceae*, order *Pirellulales*, class *Planctomycetia*, phylum *Planctomycetes*.

#### Description of *Bythopirellula polymerisocia* sp. nov.

*Bythopirellula polymerisocia* (po.ly.me.ri.so’ci.a. N.L. neut. n. *polymerum* polymer; L. fem. adj. *socia* allied, united; N.L. fem. adj. *polymerisocia*; corresponding to the characteristic of the cells to attach to polymeric material).

Cells are pear-shaped (size: 1.1 ± 0.1 × 0.7 ± 0.1 µm), form fibrous aggregates, divide by budding and feature a dimorphic life cycle. Cells have flagella as well as polar crateriform structures and fibres. Cells are mesophilic and neutrophilic chemoorganoheterotrophs, growing over a range of 20–30 °C (optimum 27 °C) and at pH 6.0–9.5 (optimum 8.5). Colonies are white. The genome size of the type strain is 6.14 Mb with a G+C content of 52.9 ± 2.2%.

The type strain is Pla144^T^ (= DSM 104841^T^ = VKM B-3442^T^), isolated from plastic waste (polyethylene) found on the banks of the river Warnow close to Rostock, Germany. The type strain genome (acc. no. SJPS00000000) and 16S rRNA gene sequence (acc. no. MK554548) are available from GenBank.

#### Description of the genus *Pseudobythopirellula* gen. nov.

*Pseudobythopirellula* (Pseu.do.by.tho.pi.rel’lu.la. Gr. adj. *pseude*s false, mendacious; N.L. fem. n. *Bythopirellula* a proposed name of a bacterial genus; N.L. fem. n. *Pseudobythopirellula*, false *Bythopirellula*).

Cells are pear-shaped and form aggregates. The distinctiveness of the genus is confirmed by phylogenetic analysis. The type species is *Pseudobythopirellula maris*. The genus belongs to the family *Lacipirellulaceae*, order *Pirellulales*, class *Planctomycetia*, phylum *Planctomycetes*.

#### Description of *Pseudobythopirellula maris* sp. nov.

*Pseudobythopirellula maris* (ma’ris. L. gen. n. *maris* of the sea; corresponding to the origin of the type strain from the sea).

Cells are pear-shaped (size: 1.3 ± 0.2 × 0.6 ± 0.1 µm), form very dense aggregates and divide by budding. Cells have crateriform structures and form much fibrous material. Cells are mesophilic and neutrophilic chemoorganoheterotrophs, growing over a range of 15–36 °C (optimum 27 °C) and at pH 5.5–9.0 (optimum 7.0). Colonies are white. The genome size of the type strain is 5.07 Mb with a DNA G+C content of 66.2 ± 0.9%.

The type strain is Mal64^T^ (= DSM 100832^T^ = LMG 29020^T^, synonym Malle64), isolated from phytoplankton collected at a public beach on Mallorca Island, Spain. The type strain genome (acc. no. SJPQ00000000) and 16S rRNA gene sequence (acc. no. MK554544) are available from GenBank.

## Electronic supplementary material

Below is the link to the electronic supplementary material.

Supplementary material 1 (DOCX 1106 kb)

Supplementary material 2 (XLSX 26 kb)

Supplementary material 3 (XLSX 18 kb)
