# Supplementary material for: Updates to the recently introduced family Lacipirellulaceae in the phylum Planctomycetes: isolation of strains belonging to the novel genera Aeoliella, Botrimarina, Pirellulimonas and Pseudobythopirellula and the novel species Bythopirellula polymerisocia and Posidoniimonas corsicana
Source: Antonie Van Leeuwenhoek. 2020 Nov 5;113(12):1979–97. doi: 10.1007/s10482-020-01486-3 (PMC7717034; doi:10.1007/s10482-020-01486-3)
Supplement: Supplementary file 1 — Supplementary material 1 (DOCX 1106 kb) [file 10482_2020_1486_MOESM1_ESM.docx]

***Supplementary Material***

**Updates to the recently introduced family *Lacipirellulaceae* in the phylum *Planctomycetes*: isolation of strains belonging to the novel genera *Aeoliella*, *Botrimarina*, *Pirellulimonas* and *Pseudobythopirellula* and the novel species *Bythopirellula polymerisocia* and *Posidoniimonas corsicana***

Sandra Wiegand^1,2^, Mareike Jogler^3^, Christian Boedeker^4^, Anja Heuer^4^, Stijn H. Peeters^2^, Nicolai Kallscheuer^2^, Mike S. M. Jetten^2^, Anne-Kristin Kaster^1^, Manfred Rohde^5^, Christian Jogler^2,3,^*

^1^Institute for Biological Interfaces 5, Karlsruhe Institute of Technology, Eggenstein-Leopoldshafen, Germany

^2^ Department of Microbiology, Radboud Universiteit, Nijmegen, The Netherlands

^3^ Department of Microbial Interactions, Friedrich Schiller University, Jena, Germany

^4^ Leibniz Institute DSMZ, Braunschweig, Germany

^5^ Central Facility for Microscopy, Helmholtz Centre for Infection Research, Braunschweig, Germany

* corresponding author: christian.jogler@uni-jena.de

Keywords: aquatic bacteria, biotic surfaces, *Lacipirellula*, *Bythopirellula goksoyri, Pirellulales, Planctomycetia*


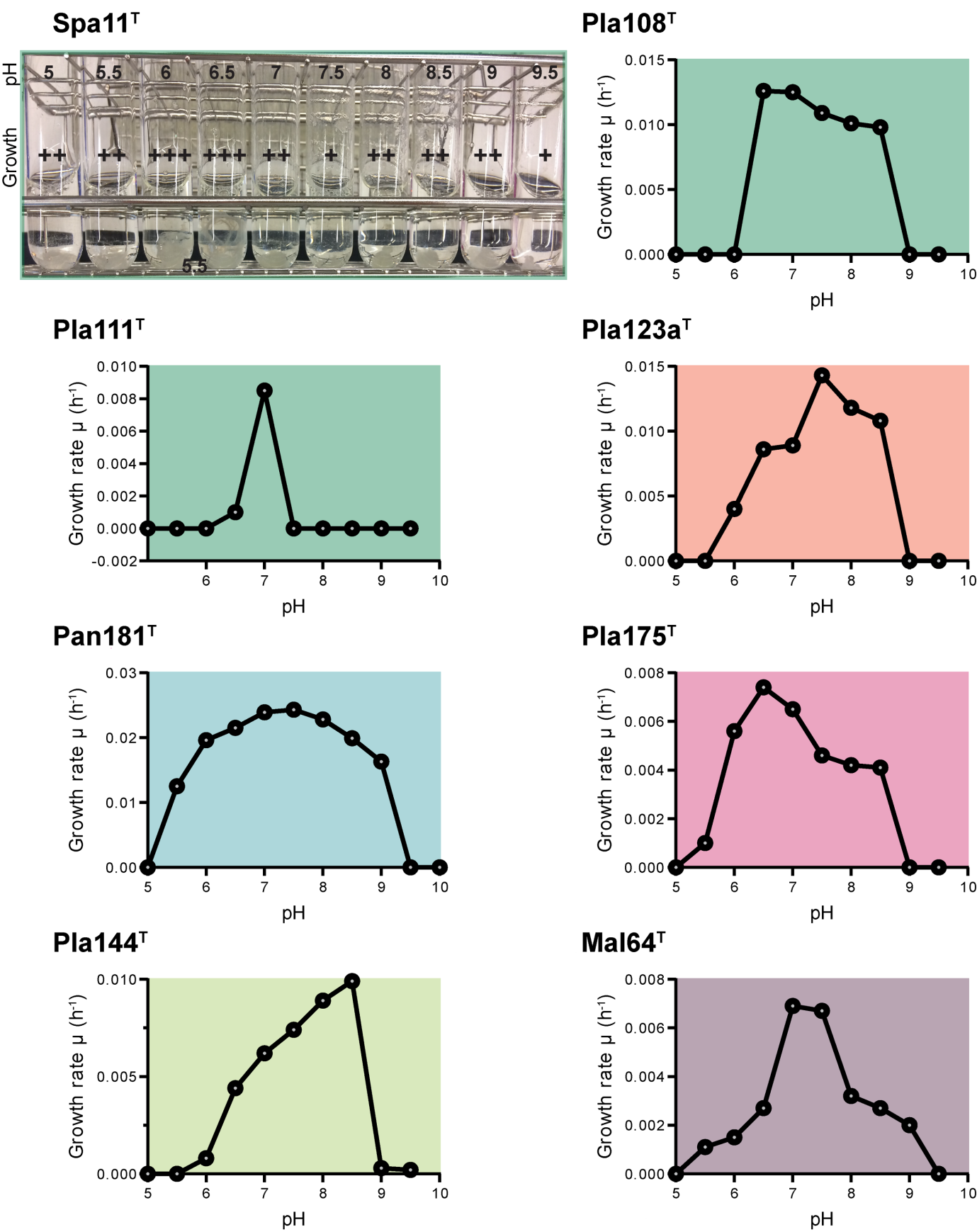


**Figure S1. Growth at different pH.** Species suspected to belong to the same species (groups I to VII) have the same colour: (I) dark green, (II) red, (III) blue, (V) hot pink, (VI) light green and (VII) purple. For strain Spa11^T^ the optical density could not be determined due to aggregate formation and therefore no growth rate could be calculated.


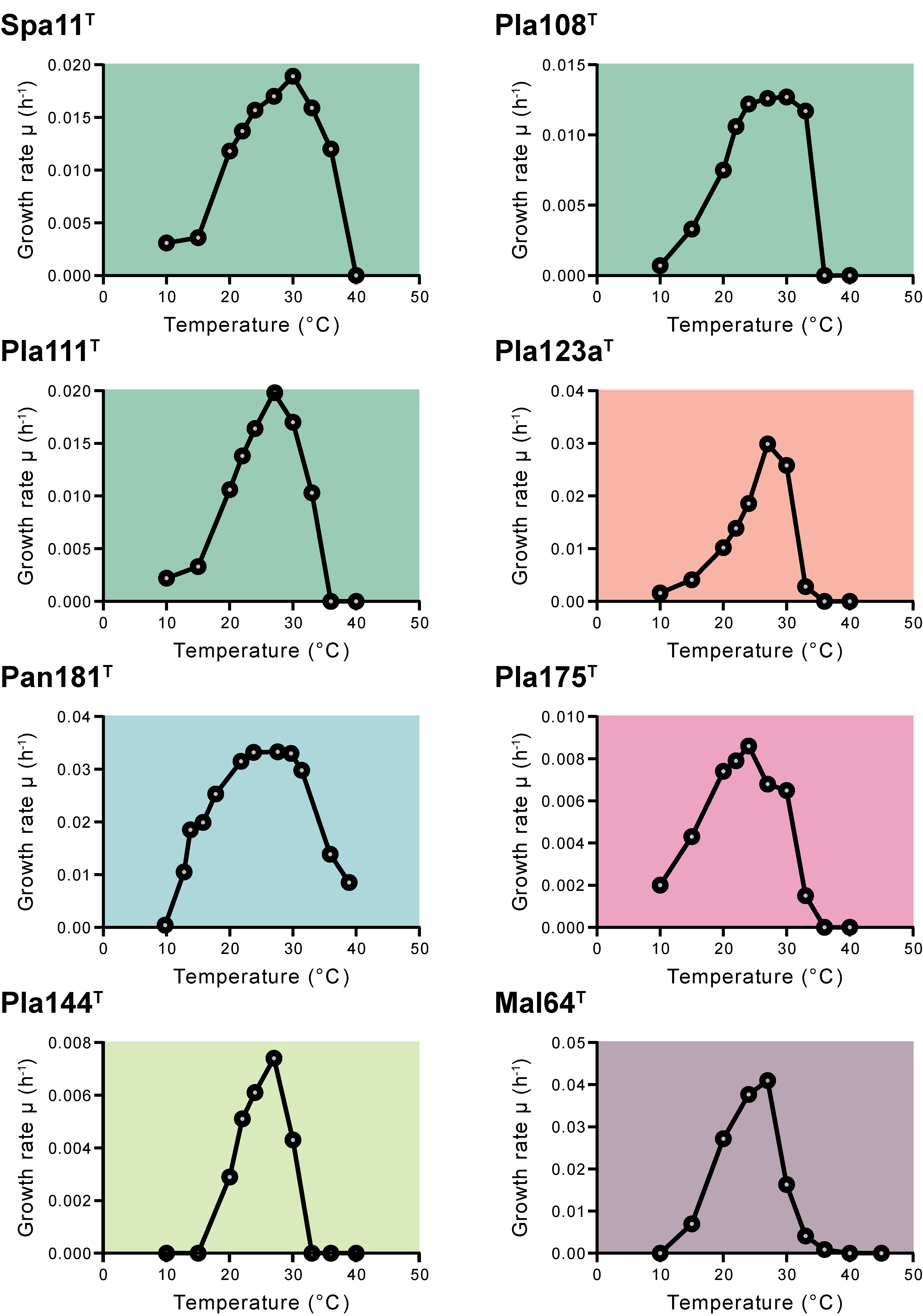


**Figure S2. Growth at different temperatures.** Species suspected to belong to the same species (groups I to VII) have the same colour: (I) dark green, (II) red, (III) blue, (V) hot pink, (VI) light green and (VII) purple.

**Table S1.** Sampling and cultivation of all novel strains.

|  |  |  |  |  |
| --- | --- | --- | --- | --- |
| **Strain** | **Collection date** | **Sampling location** | **Sampling coordinates** | **Habitat** |
| Mal64 | 2014-9-23 | S'Arenal, Island Mallorca, Spain | 39.5126 N 2.7470 E | phytoplankton close to a public beach |
| Pan181 | 2013-9-10 | Panarea Island, Italy | 38.6387 N 15.1068 E | yellow gray fringes in a hydrothermal area |
| Pla108 | 2014-9-4 | Hohe Düne, Rostock, Germany | 54.183 N 12.095 E | wood pellets stored in an incubator |
| Pla111 | 2014-9-4 | Hohe Düne, Rostock, Germany | 54.183 N 12.095 E | incubator |
| Pla123a | 2014-9-4 | Heiligendamm, Germany | 54.146 N 11.843 E | wood pellets stored in an incubator |
| Pla144 | 2014-9-4 | Unterwarnow, Rostock, Germany | 54.097 N 12.151 E | polyethylene waste found on the shore |
| Pla175 | 2014-9-4 | Unterwarnow, Rostock, Germany | 54.106 N 12.096 E | wood pellets stored in an incubator, close to the discharge of a wastewater treatment plant |
| Spa11 | 2014-5-12 | Spain: Costa Brava | 41.663 N 2.910 E | seawater from the Mediterranean Sea |

**Tables S2-S6.** Distance marker values determined for average nucleotide identity (ANI), *rpoB* gene identity and 16S rRNA gene sequence identity, amino acid identity (AAI) and percentage of conserved proteins (POCP). All values are given in percent. **Tables S2-S6 are provided in a separate Excel file.**

**Table S7. Cell sizes of the novel strains and already published species.**

| **Strain*** | **Cell length (µm)** | **Cell width (µm)** |
| --- | --- | --- |
| Spa11^T^ | 1.2±0.2 | 0.6±0.1 |
| Pla108^T^ | 1.2±0.1 | 0.6±0.1 |
| Pla111^T^ | 1.3±0.2 | 0.7±0.1 |
| *Posidoniimonas corsica* KOR34^T^ | 1.4±0.2 | 1.1±0.2 |
| Pla123a^T^ | 1.6±0.3 | 1.0±0.2 |
| Pan181^T^ | 1.4±0.3 | 0.7±0.1 |
| *Lacipirellula parvula* PX69^T^ | 0.9±0.4 | 0.5-0.9 |
| ‘*Lacipirellula limnantha*’ I41^T^ | 1.3±0.3 | 0.7±0.1 |
| Pla175^T^ | 1.4±0.2 | 0.8±0.1 |
| Pla144^T^ | 1.1±0.1 | 0.7±0.1 |
| Mal64^T^ | 1.3±0.2 | 0.6±0.1 |

** ’Bythopirellula goksoyri*’ Pr1d is not shown due to incompatible size data

**Table S8:** Analysis of primary metabolism of all strains. Different shades of blue entries indicate enzymes both capable of the same reaction and the different green shades indicate different subunits of the same enzymes. **Table S8 is provided as a separate Excel file.**
